# Efficacy and Safety of Metformin Compared with Watchful Observation for Breast Fibroadenomas: A Randomized Controlled Assessor-Blinded Study from India

**DOI:** 10.7759/cureus.112207

**Published:** 2026-07-07

**Authors:** Keshav Kumar, Kumari Pallavi, Kumar Martand, Hitesh Mishra, Sukalyan Saha Roy, Lalit Mohan, Prem Prakash, Ranjana

**Affiliations:** 1 Pharmacology and Therapeutics, Indira Gandhi Institute of Medical Sciences, Patna, IND; 2 Anatomy, Employees' State Insurance Post Graduate Institute of Medical Sciences and Research (ESI-PGIMSR), Employees' State Insurance Corporation (ESIC) Medical College, Kolkata, IND; 3 General Surgery, Indira Gandhi Institute of Medical Sciences, Patna, IND; 4 Obstetrics and Gynecology, Indira Gandhi Institute of Medical Sciences, Patna, IND

**Keywords:** breast fibroadenoma, efficacy, metformin, randomized controlled trial (rct), safety

## Abstract

Introduction: Breast fibroadenoma (FA) is the most common benign, painless, and solid mass in women of reproductive age and presents as a single or multiple masses located in the same or both breasts. No drug is approved for the treatment of breast FA as yet. Because of the estrogen-dependent and proliferative features of FA and the anti-proliferative and sex hormone-suppressing characteristics of metformin, we aimed to assess metformin for its clinical effectiveness in the treatment of breast FA. The primary objective was to evaluate the efficacy of metformin in comparison with watchful observation in reducing the number and size of breast lumps among patients of breast FA, whereas the secondary objective was to evaluate the safety of metformin used for the treatment of breast FA.

Methods: This was a hospital-based, prospective, two-arm, parallel design, assessor-blinded, randomized controlled trial. Pre-menopausal women aged 18-50 years and diagnosed with breast FA with a lump size < 5 cm were included in the study. Assessment of lump size was done under two categories: smaller mass (<2 cm) and larger mass (≥ 2 cm). One hundred and sixty-eight women were randomized into two groups in a 1:1 ratio to receive either 1000 mg of metformin daily or be put on watchful observation for 12 weeks. The study participants were followed for 24 weeks. Primary outcomes were mean change from baseline in size of the smallest and largest masses and in number of masses at 12 and 24 weeks, as well as proportion of women showing complete regression of masses at 12 and 24 weeks. Descriptive and inferential analyses were performed. Both intention-to-treat (ITT) and per protocol (PP) analyses were performed.

Results: Results of both ITT and PP analyses showed a significant difference between the two groups for mean change from baseline in size of smallest and largest masses as well as in number of masses at 12 and 24 weeks. In ITT analysis, mean change from baseline in size of largest masses, in size of smallest mass, and in number at 12 weeks were, respectively, -6.18 (±3.88), -2.10 (±2.36), and -0.70 (±1.03) in the metformin group as compared to, respectively, -3.24 (±3.40), -0.57 (±1.81), and -0.18 (±1.29) in the watchful observation group, and differences were statistically significant (p < 0.001, < 0.001, and 0.004, respectively). The same changes at 24 weeks were, respectively, -7.18 (±4.24), -2.16 (±2.53), and -1.07 (±1.10) in the metformin group as compared to -4.08 (±4.36), -1.21 (±1.64), and -0.28 (±1.49) in the watchful observation group, and the differences were statistically significant (p=0.002, 0.017, and <0.001, respectively).

Conclusion: Treatment with metformin (1000 mg twice daily) for 12 weeks resulted in a reduction in both the size and the number of breast FAs measuring < 5 cm. At 24 weeks, the effect of metformin in reducing the size and number of breast masses was carried forward post treatment.

## Introduction

Fibroadenoma (FA) is the most common benign, painless, solid mass of the female breast [1]. The incidence in India varies with regions and settings, most probably attributable to variable terminologies used for FA, and ranges between 12% and 25% [2]. FA is most commonly seen among young women between 14 and 35 years of age and is less common among postmenopausal women [3]. It may present as a solitary lump in one breast or as multiple lumps in the same or both breasts. A palpable FA is typically a firm and very mobile lump with a round border. The size of the lump is highly variable, ranging from a few millimeters to many centimeters [4].

The exact underlying etiology is yet to be determined, but the presence of estrogen receptor (ER) and progesterone receptor (PR) on breast tissues and their sensitivity to hormonal changes suggest a hormonal pathophysiology [5, 6]. Additionally, the mediator complex subunit 12 (MED 12) gene has also been implicated in the pathophysiology of FA [7]. Histologically, FA is characterized by cellular proliferation of stroma and glands [8]. The diagnosis is based on triple assessment, which encompasses three modalities: physical examination, imaging (mammography and/or ultrasonography), and biopsy (fine needle aspiration cytology (FNAC) and core biopsy) [9,10]. In the case of small FA with typical features on clinical examination and ultrasound scan, particularly in young women, biopsy of breast tissues is usually avoided [11].

For small and simple FAs, which are usually asymptomatic, watchful observation is the main approach, whereas for large and symptomatic lumps, surgical interventions like lumpectomy, excisional biopsy, and minimally invasive interventions including cryoablation, ultrasound-guided vacuum-assisted biopsy, and radiofrequency ablation can be used [5, 12]. For medical therapy, many drugs like tamoxifen [13], danazol [14], and centchroman [15] have been investigated, but none has reached an approval level. Many dietary supplements like vitamin E [16] and C [17] and evening primrose oil [18, 19] have also been evaluated for their benefit in FA. An unacceptable number of patients do not regress; rather, some patients show mass enlargement on watchful observation, and such FAs need surgical interventions. Surgical interventions have specific indications and certain limitations (being traumatic, although new minimally invasive procedures have overcome this) and are distressful to the patients [5]. This emphasizes the need for effective medical therapy, which could increase the regression rate and decrease the need for surgical interventions.

Metformin, an established first-line anti-hyperglycemic agent for type 2 diabetes mellitus, has been demonstrated to have anti-proliferative effects and anti-estrogenic properties on various tissues, including breast tissues. Because of the estrogen-dependent and proliferative features of FA and the anti-proliferative, sex hormone-suppressing characteristics of metformin, as well as its relatively low frequency of adverse effects, the clinical effectiveness of metformin on FA is being assessed [20, 21]. A randomized, placebo-controlled trial using 500 mg twice daily demonstrated significant size reduction of smaller FA with lump size <2 cm but failed to demonstrate any significant size reduction of larger FA with lump size of ≥2 cm and recommended assessment of a higher dose of metformin to see effects on larger lumps [1]. We, therefore, aimed to assess the efficacy and safety of metformin 1000 mg twice daily in patients with breast FA. The primary objective was to evaluate the efficacy of metformin in comparison with watchful observation in reducing the number and size of breast lumps among patients with breast FA, whereas the secondary objective was to evaluate the incidence and severity of treatment-emergent adverse events (TEAEs) associated with metformin used for the treatment of breast FA.

## Materials and methods

Study design, setting, and duration

This was a prospective, two-arm, parallel design, assessor-blinded, randomized controlled trial designed to compare the efficacy and safety of metformin in comparison with watchful observation among patients with breast FA. This was a hospital-based study conducted on the outpatients at the Department of General Surgery and the Department of Obstetrics and Gynecology (OB&G) in collaboration with the Department of Pharmacology at Indira Gandhi Institute of Medical Sciences, Patna, India, and was of 12 months’ duration from October 16, 2024, to October 15, 2025.

Sample size calculation

Sample size calculation was performed using the online calculator Statulator.com. The sample size calculation was based on the change in the size of the breast mass from the previous study conducted by Alipour et al. [1], wherein the mean reduction in size of the smallest breast mass in the metformin and control groups was 3.26 mm±3.26 and 0.14 mm±6.48, respectively. Taking this estimate and an anticipated clinically significant difference in the change in size of the smallest mass of 2 mm, with a two-sided alpha of 0.05, power of 90%, and a 1:1 allocation ratio in two groups, the minimum sample size required was calculated to be 141. Assuming an attrition rate of 20%, the total required sample was 168.

Inclusion criteria

Pre-menopausal women aged 18-50 years old with one or more unilateral or bilateral FAs with lump size < 5 cm and willing to participate and give written informed consent were included in the study.

Exclusion criteria

Exclusion criteria included a breast lump size ≥5 cm; pregnancy or breastfeeding; vegetarianism; body mass index (BMI) >29.9 kg/m²; a history of breast cancer; allergy to biguanides; diabetes mellitus; metabolic syndrome; renal or hepatic dysfunction; concomitant use of medications that could influence study outcomes, including oral contraceptives, estrogen- or progestin-containing medications, phytoestrogen-containing medications, gonadotropin-releasing hormone (GnRH) agonists or antagonists, clomiphene, tamoxifen, aromatase inhibitors, and danazol; and irregular medication use or complete non-compliance.

Interventions

Women in the metformin group received 1000 mg standard-release metformin tablets twice daily for 12 weeks, whereas women in the control group were put on watchful observation for self-regression for the same period. The study participants and their attendants vowed to adhere to the intervention. In order to ensure adherence to the intervention, participants were asked to take the metformin tablet in the presence of their attendants and write the time of administration in the diary given to them, and also bring the diary and strip of used tablets to the next visit.

Outcomes

Primary Outcomes 

Primary outcomes were mean change from baseline in size of smallest and largest masses as well as mean change from baseline in number of masses at 12 weeks (end of treatment) and 24 weeks (end of the study). Another primary outcome was the percentage of women with a complete absence of any detectable mass (regression rate) at 12 and 24 weeks.

Secondary Outcomes

The secondary outcome was the number (percentage) of women showing TEAEs and any serious TEAE warranting drug withdrawal at 12 weeks.

Participant enrolment

The study was conducted in six visits; Visit 1: screening visit, visit 2: randomization/baseline visit (week 0), visit 3: first follow-up visit (week 4), visit 4: second follow-up visit (week 8), visit 5: third follow-up (end of treatment) visit (week 12), and visit 6: fourth follow-up (end of study) visit (week 24). The data were collected using a study-specific case report form (CRF). At the screening visit (visit 1), participants giving written informed consent were screened as per inclusion/exclusion criteria. Eligible patients were provided with a printed patient information sheet in Hindi and English explaining the nature of FA and possible benefits and harm with metformin. Subjects meeting the inclusion criteria were enrolled in the study. Vital signs such as blood pressure, temperature, pulse rate, and respiration rate, and demographic characteristics such as age, height, weight, and BMI were recorded.

At randomization/baseline visit (visit 2 at week 0), eligible patients were randomized to receive either a metformin 1000 mg tablet twice daily (metformin group) or to be put on watchful observation (watchful observation group) for 12 weeks. Permuted block randomization with block sizes of two, four, and six was done. A randomization sequence with codes was developed online using the website www.sealedenvelope.com, and allocation was concealed until the last subject was recruited. The investigator generated the randomization sequence and enrolled patients, whereas a pharmacist who was not part of the study assigned the intervention. Being an open-label study, subjects and the investigator were aware of the intervention given. However, the sonologists who assessed the outcome were not aware of the intervention given. Vital signs such as blood pressure, temperature, pulse rate, and respiration rate; hematological parameters such as CBC, ESR, liver function test (LFT), and kidney function test (KFT); and biochemical parameters such as random blood sugar (RBS) and HbA1c were recorded. Clinical characteristics pertaining to breast FA such as number of masses (single or multiple), laterality (masses located in the left, right, or both breasts), and size of larger and smaller masses. Assessment of the number and size of breast masses was done by breast ultrasonography. Assessment of size (largest diameter on breast ultrasound scan) was done under two categories: smaller mass (<2 cm) and larger mass (≥2 cm but <5 cm). For the smaller mass category, the size of the smallest mass and for the larger mass category, the size of the largest mass were recorded.

At subsequent visits, vital signs such as blood pressure, temperature, pulse rate, and respiration rate; any TEAE; hematological parameters such as CBC, ESR, LFT, and KFT; biochemical parameters such as RBS and HbA1c; number of breast masses or whether the mass regressed completely; and size of largest and smallest masses were recorded as usual. TEAE were defined as any adverse event that first occurs or worsens in severity after the administration of the first dose of the investigational product (or study treatment), regardless of whether it is considered related to the treatment. 

Ethical consideration

The study protocol was approved by the Institutional Ethics Committee (IEC), Indira Gandhi Institute of Medical Sciences, Patna, India (Ref. No. 1440/IEC/IGIMS/2024, dated 04/04/2024), and registered prospectively with the Clinical Trials Registry-India (Ref. No. CTRI/2024/08/072768, dated 20/08/2024). The study was conducted following Good Clinical Practice in accordance with the principles of the Declaration of Helsinki. 

Statistical analysis

Data were entered in a Microsoft Excel spreadsheet (Microsoft Corporation, Redmond, WA, USA) and transferred to the data analysis software Jamovi (version 2.6.26; The jamovi project, Sydney, Australia) for analysis. Missing data was managed by imputation of the last observation. Data were checked for normal distribution by visual inspection of Q-Q plots and histograms and statistically using the Shapiro-Wilk test. The change in the number of masses and the change in the size of the larger and smaller masses were near normally distributed or skewed to one side (left or right). Considering the central limit theorem, we applied a parametric test to even skewed data because the sample size was adequate. Homogeneity of variance was checked using Levene’s test and was found to be statistically insignificant (>0.05). Descriptive and inferential analyses were performed. Continuous data were presented as mean±SD, and categorical data were presented as number with percentage. For continuous data, intergroup comparison was performed using an independent t-test. Categorical data were analyzed using the chi-square/Fisher exact test. Both intention-to-treat (ITT) and per protocol (PP) analyses were performed. A p-value of <0.05 was considered for statistical significance.

## Results

**Figure 1:**
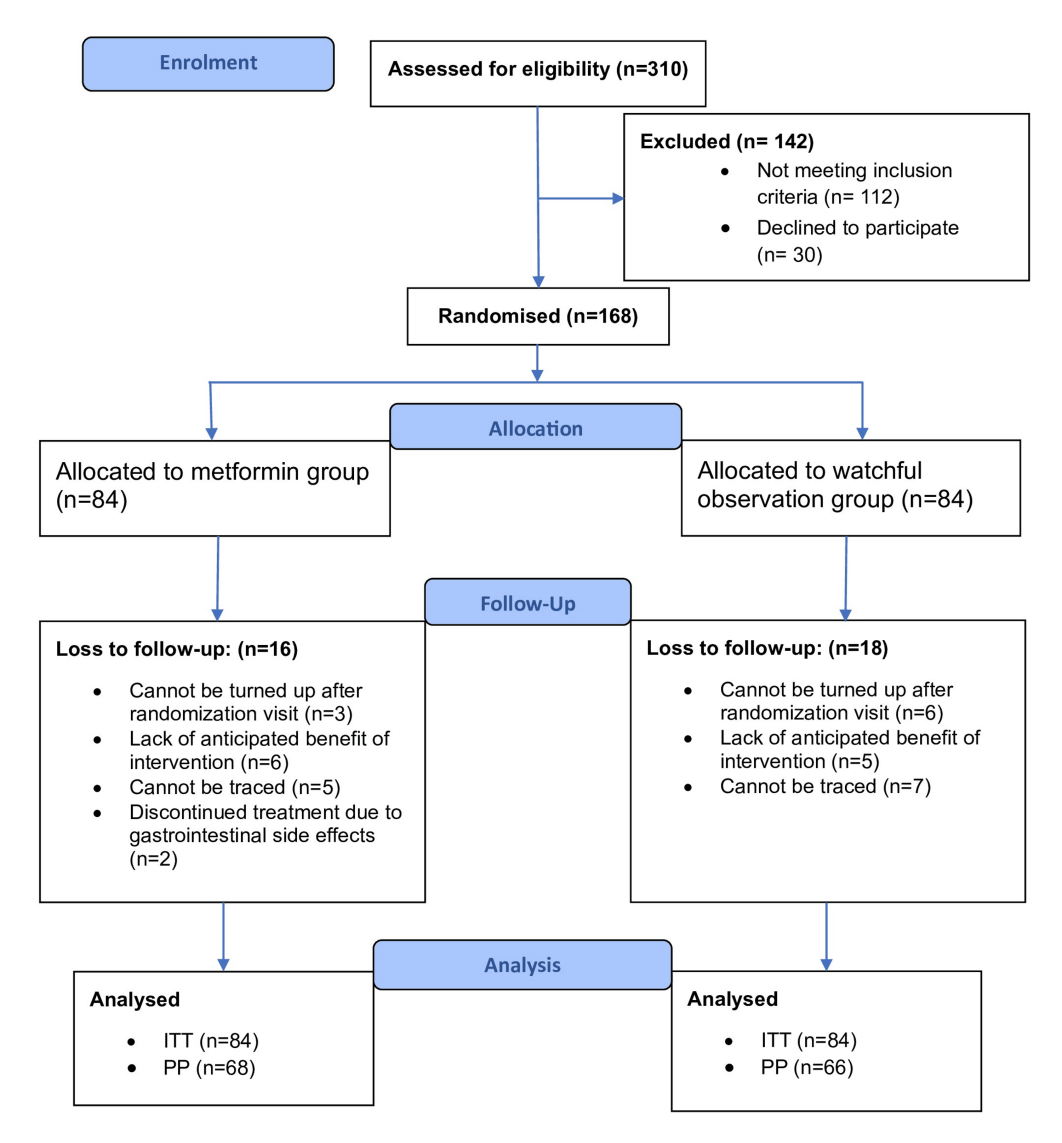
CONSORT flow diagram showing number of study participants involved in enrolment, allocation, loss to follow-up and analysis CONSORT: Consolidated Standards of Reporting Trials; ITT: intention-to-treat; PP: per protocol

To achieve the sample size of 168, we screened 310 patients for inclusion and exclusion criteria. Out of 142 which were excluded during screening, 112 patients did not meet the inclusion criteria, and 30 patients declined to participate. Eighty-four patients were allocated to each group. In the metformin group, three patients did not turn up at the randomization visit, six discontinued intervention due to lack of anticipated benefit, five could not be traced, and two discontinued intervention due to intolerability and gastrointestinal side effects. In the watchful observation group, six patients could not turn up at the randomization visit, five discontinued the intervention due to lack of anticipated benefit, and seven could not be traced. A Consolidated Standards of Reporting Trials (CONSORT) flow diagram showing screening, inclusion, exclusion, and allocation of study participants and their discontinuation or loss to follow-up with reasons and number of participants available for data analysis is provided with Figure 1.

There were no significant differences between the two groups for demographic characteristics such as age, weight, and height; biochemical parameters such as BMI, RBS, and HbA1c; and clinical characteristics such as number of breast masses, size of the largest masses, and size of the smallest masses (Table 1). At baseline visits, 38 women in both the metformin and watchful observation groups had larger breast masses (size ≥ 2 cm), whereas 63 women in the metformin group and 58 women in the watchful observation group had smaller breast masses (size < 2 cm).

**Table 1:** Baseline demographic, biochemical and clinical characteristics of study participants *Independent t-test; BMI-body mass index; RBS-random blood sugar

Characteristics	Group	N	Mean	Median	SD	p-value*	t-statistic
Age (years)	Metformin	84	26.32	24.00	6.69	0.851	0.188
	Watchful observation	84	26.13	24.00	6.44		
Weight (kg)	Metformin	84	56.16	55.80	5.14	0.879	0.153
	Watchful observation	84	56.03	55.30	5.46		
Height (m^2^)	Metformin	84	1.58	1.58	0.06	0.293	-1.056
	Watchful observation	84	1.59	1.58	0.07		
BMI (kg/m^2^)	Metformin	84	22.61	22.75	1.84	0.249	1.158
	Watchful observation	84	22.28	22.10	1.89		
RBS (mg/dL)	Metformin	84	132.69	130.00	16.23	0.232	1.199
	Watchful observation	84	130.10	129.50	11.42		
HbA1c (%)	Metformin	84	5.55	5.60	0.44	0.600	0.525
	Watchful observation	84	5.52	5.60	0.38		
Number of masses	Metformin	84	2.02	1.00	1.35	0.956	0.055
	Watchful observation	84	2.01	1.00	1.44		
Size of the largest mass (mm)	Metformin	38	26.74	24.00	6.41	0.975	-0.031
	Watchful observation	38	26.79	23.00	8.29		
Size of smallest mass (mm)	Metformin	63	9.68	8.00	4.69	0.581	0.554
	Watchful observation	58	9.24	8.00	4.01		

**Figure 2:**
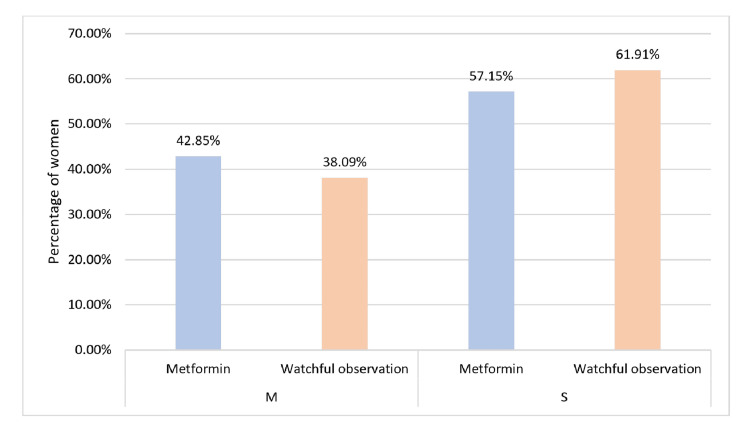
Baseline frequency of types of breast masses M: multiple; S: single

In the metformin group, 42.85% (36) of women had multiple masses, and 57.15% (48) of women had single masses, whereas in the group put on watchful observation, 38.09% (32) of women had multiple masses, and 61.91% (52) of women had single masses (Table 2 and Figure 2).

**Table 2:** Baseline frequency of types of breast masses

Lesion type	Group	Counts	Percentage of group total (%)
Multiple	Metformin	36	42.85%
	Watchful observation	32	38.09%
Single	Metformin	48	57.15%
	Watchful observation	52	61.91%

**Figure 3:**
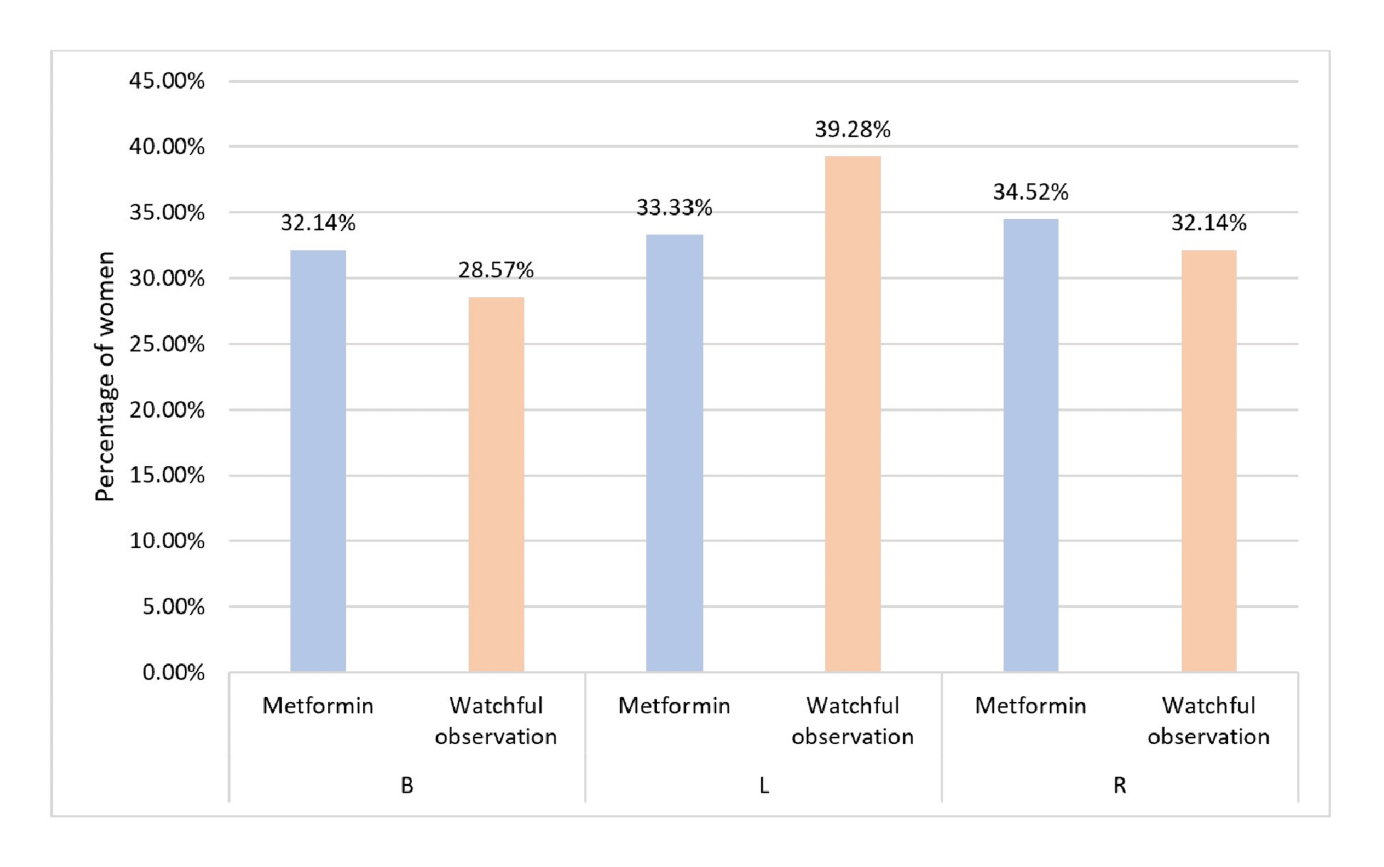
Baseline frequency of laterality of breast masses B: bilateral; L: left; R: right

Laterality (located in the left or right or both breasts) of breast masses is presented in Table 3 and depicted in Figure 3. In the metformin group, 32.14% (27) of women had masses in both breasts (bilateral), 33.33% (28) had masses in their left breasts, and 34.52% (29) had masses in their right breasts, whereas in the group put on watchful observation, 28.57% (24) of women had masses in both breasts (bilateral), 39.28% (33) had masses in their left breasts, and 32.14% (27) had masses in their right breasts.

**Table 3:** Baseline frequency of laterality of breast masses

Laterality	Group	Counts	Percentage of group total (%)
Bilateral	Metformin	27	32.14%
	Watchful observation	24	28.57%
Left	Metformin	28	33.33%
	Watchful observation	33	39.28%
Right	Metformin	29	34.52%
	Watchful observation	27	32.14%

Primary outcomes

At 12 weeks, 30 women in the metformin group and 28 women in the watchful observation group had larger masses, whereas 51 women in the metformin group and 46 women in the watchful observation group had smaller masses. At 24 weeks, 26 women in the metformin group and 24 women in the watchful observation group had larger masses, whereas 48 women in the metformin group and 45 women in the watchful observation group had smaller masses.

Change in Size of the Largest and Smallest Masses

**Figure 4:**
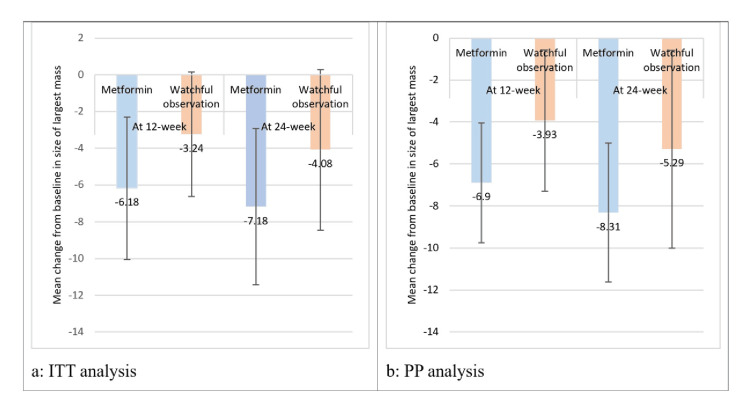
Comparison between two groups for change from baseline in size of largest masses at 12 and 24 weeks (panel a: ITT analysis; panel b: PP analysis) ITT: intention-to-treat; PP: per protocol

The result of the ITT analysis showed that the mean change from baseline in the size of the largest mass at 12 weeks was -6.18 (±3.88) in the metformin group as compared to -3.24 (±3.40) in the watchful observation group, and the difference was statistically significant (p<0.001, t-statistic: -3.522). The same change at 24 weeks was -7.18 (±4.24) in the metformin group as compared to -4.08 (±4.36) in the watchful observation group, and the difference was statistically significant (p=0.002, t-statistic: -3.148) (Table 4, Figure 4a). In PP analysis, the mean change from baseline in the size of the largest mass at 12 weeks was -6.90 (±2.84) in the metformin group as compared to -3.93 (±3.37) in the watchful observation group, and the difference was statistically significant (p<0.001, t-statistic: -3.640). The same change at 24 weeks was -8.31 (±3.30) in the metformin group as compared to -5.29 (±4.71) in the watchful observation group, and the difference was statistically significant (p=0.011, t-statistic: -2.639) (Table 4, Figure 4b).

**Table 4:** Size and change from baseline in size of the largest and smallest breast masses *Independent t-test; ITT: intention-to-treat; PP: per protocol

Parameters	Group	ITT analysis					PP analysis				
		N	Mean	SD	p-value*	t-statistic	N	Mean	SD	p-value*	t-statistic
Baseline size of the largest mass (mm)	Metformin	38	26.74	6.41	0.975	-0.031	38	26.74	6.41	0.975	-0.031
	Watchful observation	38	26.79	8.29			38	26.79	8.29		
Baseline size of the smallest mass (mm)	Metformin	63	9.68	4.69	0.581	0.554	63	9.68	4.69	0.581	0.554
	Watchful observation	58	9.24	4.01			58	9.24	4.01		
Size of the largest mass at 12 weeks (mm)	Metformin	38	20.55	6.84	0.072	-1.827	30	19.27	6.29	0.061	-1.915
	Watchful observation	38	23.55	7.46			28	22.71	7.40		
Size of the smallest mass at 12 weeks (mm)	Metformin	63	7.59	4.65	0.191	-1.315	51	6.45	3.91	0.024	-2.290
	Watchful observation	58	8.67	4.40			46	8.46	4.72		
Size of the largest mass at 24 weeks (mm)	Metformin	38	19.55	7.25	0.055	-1.949	26	17.46	6.46	0.055	-1.966
	Watchful observation	38	22.71	6.87			24	21.00	6.25		
Size of the smallest mass at 24 weeks (mm)	Metformin	63	7.52	4.66	0.517	-0.649	48	6.44	3.92	0.130	-1.530
	Watchful observation	58	8.03	3.92			45	7.71	4.12		
Change from baseline in size of the largest mass at 12 weeks (mm)	Metformin	38	-6.18	3.88	<0.001	-3.522	30	-6.90	2.84	<0.001	-3.640
	Watchful observation	38	-3.24	3.40			28	-3.93	3.37		
Change from baseline in size of the smallest mass at 12 weeks (mm)	Metformin	63	-2.10	2.36	<0.001	-3.968	51	-2.55	2.40	<0.001	-4.470
	Watchful observation	58	-0.57	1.81			46	-0.54	1.96		
Change from baseline in size of the largest mass at 24 weeks (mm)	Metformin	38	-7.18	4.24	0.002	-3.148	26	-8.31	3.30	0.011	-2.639
	Watchful observation	38	-4.08	4.36			24	-5.29	4.71		
Change from baseline in size of the smallest mass at 24 weeks (mm)	Metformin	63	-2.16	2.53	0.017	-2.429	51	-2.94	2.91	0.002	-3.170
	Watchful observation	58	-1.21	1.64			45	-1.36	1.76		

**Figure 5:**
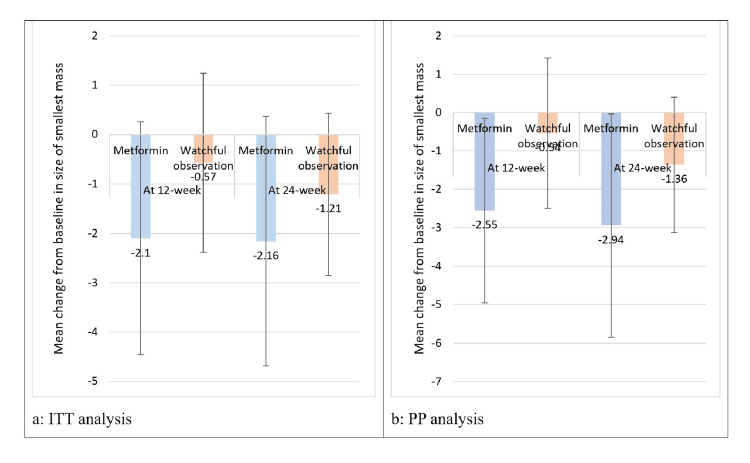
Comparison between two groups for change from baseline in size of smallest masses at 12 and 24 weeks (panel a: ITT analysis; panel b: PP analysis) ITT: intention-to-treat; PP: per protocol

The result of the ITT analysis showed that the mean change from baseline in the size of the smallest mass at 12 weeks was -2.10 (±2.36) in the metformin group as compared to -0.57 (±1.81) in the watchful observation group, and the difference was statistically significant (p<0.001, t-statistic: -3.968). The same change at 24 weeks was -2.16 (±2.53) in the metformin group as compared to -1.21 (±1.64) in the watchful observation group, and the difference was statistically significant (p=0.017, t-statistic: -2.429) (Table 4, Figure 5a). In PP analysis, the mean change from baseline in the size of the smallest mass at 12 weeks in the metformin group was -2.55 (±2.40) compared with -0.54 (±1.96) in the watchful observation group, and the difference was statistically significant (p<0.001, t-statistic: -4.470). The same change at 24 weeks was -2.94 (±2.91) in the metformin group compared with -1.36(±1.76) in the watchful observation group, and the difference was statistically significant (p=0.002, t-statistic: -3.170) (Table 4, Figure 5b).

Change in the Number of Masses

**Figure 6:**
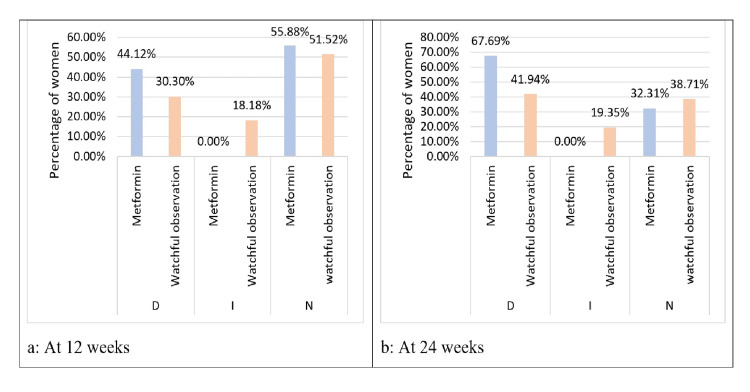
Fate of breast masses at 12 weeks (panel a) and 24 weeks (panel b) D: decrease; I: increase; N: no change

At 12 weeks, data on the number of breast masses were available from 68 women in the metformin group and 66 women in the watchful observation group. Breast masses decreased, increased, and showed no change in 44.12% (30), 0% (0), and 55.88% (38) women, respectively, in the metformin group and in 30.30% (20), 18.18% (12), and 51.52% (34) women, respectively, in the watchful observation group (Table 5, Figure 6a). At 24 weeks, data were available from 65 women in the metformin group and 62 women in the watchful observation group. Breast masses decreased, increased, and showed no change in 67.69% (44), 0% (0), and 32.31% (21) women, respectively, in the metformin group and in 41.94% (26), 19.35% (12), and 38.71% (24) women, respectively, in the watchful observation group (Table 5, Figure 6b).

**Table 5:** Fate of breast masses at 12 and 24 weeks

Fate	Group	At 12 weeks		At 24 weeks	
		Counts	Percentage of group total (%)	Counts	Percentage of group total (%)
Decrease	Metformin	30	44.12%	44	67.69%
	Watchful observation	20	30.30%	26	41.94%
Increase	Metformin	0	0.0%	0	0.0%
	Watchful observation	12	18.18%	12	19.35%
No change	Metformin	38	55.88%	21	32.31%
	Watchful observation	34	51.52%	24	38.71%

At 12 weeks, breast masses regressed completely in 26.19% (22) of women in the metformin group as compared to 11.90% (10) of women in the watchful observation group, and the difference in regression rate was statistically significant (p=0.020, χ²-statistic: 5.45). At 24 weeks, breast masses regressed completely in 43.08% (28) of women in the metformin group as compared to 24.19% (15) of women in the watchful observation group, and the difference in regression rate was statistically significant (p=0.023, χ²-statistic: 5.18) (Table 6).

**Table 6:** Regression of breast fibroadenoma # χ²: chi-square test for two independent proportions

Regression rate								
Intervention	% (Counts)	χ²-statistic	df	p-value#	% (Counts)	χ²-statistic	df	p-value#
Metformin	26.19% (22)	5.45	1	0.020	43.08% (28)	5.18	1	0.023
Watchful observation	11.90% (10)				24.19% (15)			

**Figure 7:**
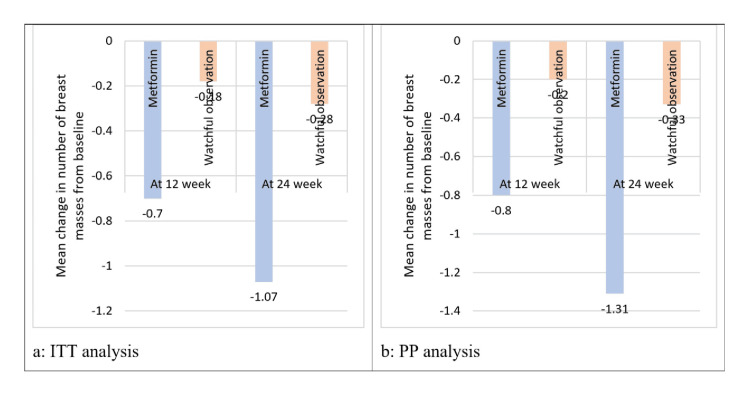
Comparison between two groups for mean change from baseline in the number of breast masses at 12 and 24 weeks (panel a: ITT analysis, panel b: PP analysis) ITT: intention-to-treat; PP: per protocol

The result of the ITT analysis showed that the mean change from baseline in the number of masses at 12 weeks was -0.70 (±1.03) in the metformin group as compared to -0.18 (±1.29) in the watchful observation group, and the difference was statistically significant (p=0.004, t-statistic: -2.910). The same change at 24 weeks was -1.07 (±1.10) in the metformin group compared to -0.28 (±1.49) in the watchful observation group, and the difference was statistically significant (p<0.001, t-statistic: -3.901) (Table 7, Figure 7a). In PP analysis, the mean change from baseline in the number of masses at 12 weeks was -0.80 (±1.08) in the metformin group compared to -0.20 (±1.45) in the watchful observation group, and the difference was statistically significant (p=0.006, t-statistic: -2.775). The same change at 24 weeks was -1.31 (±1.10) in the metformin group compared to -0.33 (±1.72) in the watchful observation group, and the difference was statistically significant (p<0.001, t-statistic: -3.801) (Table 7, Figure 7b).

**Table 7:** Comparison between two groups for the number of breast masses at different time points and change from baseline in the number of breast masses at 12 and 24 weeks *Student’s independent t-test; ITT: intention-to-treat; PP: per protocol

Characteristics	Group	ITT analysis					PP analysis				
		N	Mean	SD	P-value*	t- statistic	N	Mean	SD	P-value*	t- statistic
Baseline number of masses	Metformin	84	2.02	1.35	0.956	0.055	84	2.02	1.35	0.956	0.055
	Watchful observation	84	2.01	1.44			84	2.01	1.44		
Number of masses at 12 weeks	Metformin	84	1.32	1.30	0.021	-2.339	68	1.21	1.24	0.007	-2.753
	Watchful observation	84	1.83	1.53			66	1.88	1.57		
Number of masses at 24 weeks	Metformin	84	0.95	1.20	<0.001	-3.632	65	0.70	0.94	<0.001	-4.669
	Watchful observation	84	1.73	1.54			62	1.76	1.63		
Change from baseline in number of masses at 12 weeks	Metformin	84	-0.70	1.03	0.004	-2.910	68	-0.80	1.08	0.006	-2.775
	Watchful observation	84	-0.18	1.29			66	-0.20	1.45		
Change from baseline in number of masses at 24 weeks	Metformin	84	-1.07	1.10	<0.001	-3.901	65	-1.31	1.10	<0.001	-3.801
	Watchful observation	84	-0.28	1.49			62	-0.33	1.72		

Secondary outcomes

Incidence and severity of TEAEs

In the metformin group, 22 out of 68 women who completed the intervention developed TEAEs; none of them were serious or led to the death of a study participant, and 2 (diarrhea and abdominal pain) of them led to withdrawal of the medication. Various TEAs were decreased appetite, nausea, bloating, abdominal discomfort/pain, and diarrhea, reported in 17 (20.2%), 12 (14.3%), 10 (11.9%), six (7.1%), and two (2.4%), respectively (Table 8).

**Table 8:** Adverse events until end of intervention TEAE: Treatment emergent adverse event

Parameters	Metformin (n=65) n (%)
a. Adverse events (AEs)	
Any TEAE	22 (26.2)
Any serious TEAE	0
Any TEAE leading to study drug withdrawal	2 (2.4)
Any TEAE leading to death	0
b. Classes of TEAE	
Decreased appetite	17 (20.2)
Nausea	12 (14.3)
Bloating	10 (11.9)
Abdominal discomfort/pain	6 (7.1)
Diarrhoea	2 (2.4)

## Discussion

The result of ITT analysis showed that the mean change from baseline in size of the largest mass at 12 weeks was -6.18 (±3.88) in the metformin group as compared to -3.24 (±3.40) in the watchful-observation group, and the difference was statistically significant (p<0.001). Furthermore, the mean change from baseline in the size of the smallest mass at 12 weeks was -2.10 (±2.36) in the metformin group as compared to -0.57 (±1.81) in the watchful observation group, and the difference was statistically significant (p < 0.001) (Table 4, Figures 4a, 5a). This suggested that treatment with metformin 1000 mg twice daily for 12 weeks caused a significant reduction in the size of large as well as small breast FAs. The findings of mean change from baseline in size of the smallest mass at 12 weeks were in alignment, whereas the findings of mean change from baseline in size of the largest mass at 12 weeks were in contrast to those of the study by Alipour et al. [1]. The possible reason for the effectiveness of metformin for both smaller and larger masses could be the use of a 1000 mg twice-daily dose in this study, in contrast to the use of 500 mg twice daily in the previous study mentioned above. This suggests that metformin in patients with breast FA probably works in a dose-dependent manner to reduce the size of breast masses.

During the 12-week intervention period, breast mass size decreased in 44.12% (30) of women, remained unchanged in 55.88% (38), and increased in none of the women in the metformin group. In the watchful observation group, the corresponding proportions were 30.30% (20), 51.52% (34), and 18.18% (12), respectively (Table 5, Figure 6a). Thus, in women with metformin treatment, no further increase in the number of breast masses took place. This finding was in contrast to that of the study conducted by Alipour et al. [1], which found an increase in the number of masses in five out of 83 women in the metformin group. This contrasting finding of the present study may be attributed to the use of a high dose of metformin, which probably has a preventive role in the development of new breast FAs.

At 12 weeks, the proportion of women experiencing complete disappearance of detectable breast masses (regression rate) was 26.19% in the metformin group as compared to 11.90% in the watchful observation group, and this difference was statistically significant (p=0.020) (Table 6). This suggests metformin enhances the regression of breast FA. This finding is not in the alignment of the study by Alipour et al. [1], which found no difference in the regression of breast masses between the two groups. In ITT analysis, mean change from baseline at 12 weeks in the number of breast masses was -0.70 (±1.03) in the metformin group as compared to -0.18 (±1.29) in the watchful observation group, and the difference between the two groups was statistically significant (p=0.004) (Table 7 and Figure 7a), suggesting that metformin carried out a significant reduction in the number of breast masses.

Furthermore, the result of ITT analysis showed that the mean change from baseline in size of the largest mass at 24 weeks was -7.18 (±4.24) in the metformin group as compared to -4.08 (±4.36) in the watchful observation group, and the difference was statistically significant (p=0.002). The mean change from baseline in the size of the smallest mass at 24 weeks was -2.16 (±2.53) in the metformin group as compared to -1.21 (±1.64) in the watchful observation group, and the difference was statistically significant (p=0.017) (Table 4, Figures 4a, 5a). This suggested that the effect of treatment in causing a reduction in the size of both the largest and smallest masses was carried forward post treatment. Breast masses decreased, remained unchanged, and increased in 67.69% (44), 32.31% (21), and 0% of the women, respectively, in the metformin group as compared to 41.94% (26), 38.71% (24), and 19.35% (12) of women, respectively, in the watchful observation group (Table 5, Figure 6b). Thus, in women with metformin treatment, no further increase in the number of breast masses took place during the follow-up period of 12 weeks from 12-week to 24-week time points, again suggesting metformin probably has a preventive role in the development of new breast FA.

At 24 weeks, 43.08% (28) of women in the metformin group, as compared to 24.19% (15) of women in the watchful observation group, showed complete disappearance of breast masses, and the difference in regression rate between the two groups was statistically significant (p=0.029) (Table 6). This suggested that the effect of metformin in causing increased regression of breast FA was carried forward post treatment. In ITT analysis, mean change from baseline at 24 weeks in number of breast masses was -1.07 (±1.10) in the metformin group as compared to -0.28 (±1.49) in the watchful observation group, and the difference between the two groups was statistically significant (p<0.001) (Table 7 and Figure 7a), suggesting that the effect of metformin in causing reduction in number of breast masses was carried forward post treatment.

Cellular proliferation of stroma and glands is the key histopathologic change in breast FA. Immunohistochemistry reveals expression of ER in FA tissues [4, 5]. In vitro studies have demonstrated metformin to have anti-proliferative and growth inhibitory effects mediated through fatty acid synthesis, activation of AMP-activated protein kinase, reduction of mitochondrial metabolites, amplified apoptosis, reduced colony formation, cell cycle arrest at the G1 checkpoint, inhibition of mTOR, and decreased expression of E2F1 and cyclin D1 [22-28]. These effects have been seen in both estrogen-positive and -negative breast cancer [29]. Also, metformin has anti-estrogenic features that have made it an effective adjunct in the management of some ovarian functions and reproductive disorders, such as polycystic ovarian disease and estrogen-dependent infertility [30]. Thus, the effects of metformin in reducing the number and size of breast FAs may be attributed to its anti-proliferative and anti-estrogenic effects on breast FA tissues.

Limitations

This study has many limitations. First and foremost is the open-label design, as blinding of the investigator and study participants was not possible. The findings of this study cannot be generalized to male breast FA cases, only a few of which have been reported. We also used breast ultrasound reports from outside the hospitals, which might have introduced interobserver variability. Sample size calculation for this study was based on the size of smaller masses (<2 cm), but smaller masses were not present in all women; therefore, the findings of this study might be underpowered. In the cases where the breast mass regressed completely, we used the last data carried forward for analysis, which might have introduced some kind of deviation from true findings. Since a limited number of studies are available so far, we could compare our findings with only a few studies.

## Conclusions

Compared with watchful observation, treatment with metformin (1000 mg twice daily) for 12 weeks significantly reduced the size of both the largest and the smallest FAs, as well as the total number of breast FAs, in women with FAs measuring <5 cm. Metformin was also associated with a higher proportion of women experiencing a reduction in the number of FAs, a lower proportion with no change, and no cases of an increase in the number of FAs. These findings suggest that metformin may be an effective treatment option for women with breast FAs measuring <5 cm. Therefore, metformin may be considered protective against the development of new breast masses. The regression rate of breast FA was significantly higher in the metformin group than in the watchful observation group. Therefore, from a regression point of view, metformin treatment as compared to watchful observation can be a better option for female breast FA of size < 5 cm.

At the end of the 24-week study, there was a significantly greater reduction in the size of both the largest and the smallest masses and in the number of masses in the metformin group than in the watchful observation group. Also, there was a significantly higher regression rate of breast masses in the metformin group than in the watchful observation group. Therefore, we concluded that the effect of metformin treatment on breast FA was carried forward post treatment.
